# Phenological asynchrony between host plant and gypsy moth reduces insect gut microbiota and susceptibility to *Bacillus thuringiensis*

**DOI:** 10.1002/ece3.2460

**Published:** 2016-09-22

**Authors:** Vyacheslav V. Martemyanov, Irina A. Belousova, Sergey V. Pavlushin, Ivan M. Dubovskiy, Nikita I. Ershov, Tatyana Y. Alikina, Marsel R. Kabilov, Victor V. Glupov

**Affiliations:** ^1^ Laboratory of Ecological Parasitology Institute of Systematics and Ecology of Animals Siberian Branch Russian Academy of Sciences Novosibirsk Russia; ^2^ Biological Institute National Research Tomsk State University Tomsk Russia; ^3^ Institute of Biology Irkutsk State University Irkutsk Russia; ^4^ Laboratory of Insect Pathology Institute of Systematics and Ecology of Animals Siberian Branch Russian Academy of Sciences Novosibirsk Russia; ^5^ Institute of Cytology and Genetics Siberian Branch Russian Academy of Sciences Novosibirsk Russia; ^6^ Genomics Core Facility Institute of Chemical Biology and Fundamental Medicine Siberian Branch Russian Academy of Sciences Novosibirsk Russia

**Keywords:** 16S rRNA, insect immunity, metagenomics, phenological mismatch, tritrophic interactions

## Abstract

The phenological synchrony between the emergence of overwintering herbivorous insects and the budding of host plants is considered a crucial factor in the population dynamics of herbivores. However, the mechanisms driving the interactions between the host plant, herbivores, and their pathogens are often obscure. In the current study, an artificially induced phenological asynchrony was used to investigate how the asynchrony between silver birch *Betula pendula* and gypsy moth *Lymantria dispar* affects the immunity of the insect to bacteria, its susceptibility to the entomopathogenic bacteria *Bacillus thuringiensis*, and the diversity in its midgut microbiota. The lysozyme‐like activity in both the midgut and hemolymph plasma and the nonspecific esterase activity and antimicrobial peptide gene expression in the midgut were studied in both noninfected and *B. thuringiensis‐*infected larvae. Our results provide the first evidence that phenologically asynchronous larvae are less susceptible to *B. thuringiensis* infection than phenologically synchronous larvae, and our results show that these effects are related to the high basic levels and *B. thuringiensis*‐induced levels of lysozyme‐like activities. Moreover, a 16S rRNA analysis revealed that dramatic decreases in the diversity of the larval gut bacterial consortia occurred under the effect of asynchrony. Larvae infected with *B. thuringiensis* presented decreased microbiota diversity if the larvae were reared synchronously with the host plant but not if they were reared asynchronously. Our study demonstrates the significant effect of phenological asynchrony on innate immunity‐mediated interactions between herbivores and entomopathogenic bacteria and highlights the role of nonpathogenic gut bacteria in these interactions.

## 1 Introduction

Ecologists consider the phenological synchrony between the emergence of overwintering herbivorous insects and the budding of their host plants to represent one of the crucial factors in the population dynamics of herbivores (van Asch & Visser, 2007; Feeny, 1970; Foster, Townsend, & Mladenoff, 2013; Hunter & Lechowicz, 1992; Ivashov, Boyko, & Simchuk, 2002; Martemyanov, Pavlushin, Dubovskiy, Belousova et al., 2015; Martemyanov, Pavlushin, Dubovskiy, Yushkova, et al., 2015). If the larvae hatch prior to the host plant budburst, the larvae may starve, whereas if the larvae hatch too late, the foliage quality may be reduced (reviewed in van Asch & Visser, 2007 and references therein), although the occurrence of this phenomenon is related to studied species (Kharouba, Vellend, Sarfraz, & Myers, 2015) and global climate processes (Uelmen et al., 2016). This issue has been studied for more than 40 years (Feeny, 1970; Foster et al., 2013; Hunter & Elkinton, 1999, 2000; Hunter & Lechowicz, 1992; Ivashov et al., 2002; Martemyanov, Pavlushin, Dubovskiy, Yushkova, et al., 2015). However, a limited number of studies have demonstrated how insects and herbivores interact within a tritrophic chain under such synchrony and few have investigated the effects of both top‐down and bottom‐up forces (Hunter & Elkinton, 1999, 2000; Martemyanov, Pavlushin, Dubovskiy, Yushkova, et al., 2015). We recently showed that phenological asynchrony between *Betula pendula* Roth. trees and *Lymantria dispar* L. larvae (an eruptive defoliator related to the spring feeding guild) leads to decreases in herbivore fitness and significant increases in larval susceptibility to baculoviral disease (Martemyanov, Pavlushin, Dubovskiy, Yushkova, et al., 2015). Moreover, the activation of asymptotic baculoviral infection (covert infection) in an overt form affected approximately 60% of virus‐carrying insects if the larvae were reared asynchronously with the host plant. Because baculoviruses can induce spontaneous epizootics in the insect population (Cory & Myers, 2003), we considered the phenological asynchrony between host plants and insect defoliators to represent a predictor of natural viral epizootics. The modification of insect host innate immunity parameters under the effect of asynchrony has been reported to be one of the mechanisms that drives insect susceptibility to baculoviruses (Martemyanov, Pavlushin, Dubovskiy, Yushkova, et al., 2015). In addition, dramatic changes in leaf quality during the first 20 days of development have been suggested to represent the main mode of action of asynchrony on insect antiviral immunity (Chernyak et al., 2016; Martemyanov, Pavlushin, Dubovskiy, Yushkova, et al., 2015). However, the effect of asynchrony on insect bacterial diseases, such as intestinal bacteria *Bacillus thuringiensis*, which is widely used for the pest management (Garczynski & Siegel, 2007), remains unclear because different insect immune parameters are affected in different ways by the same trophic factor (Martemyanov, Dubovskiy, Belousova, et al., 2012; Martemyanov, Dubovskiy, Rantala, et al., 2012; Martemyanov et al., 2013; Martemyanov, Pavlushin, Dubovskiy, Belousova et al., 2015). Therefore, in this study, we tested how phenological asynchrony would affect the interaction between *L. dispar* and *B. thuringiensis* Berliner and investigated the mechanisms underlying this interaction.

*Bacillus thuringiensis* is a Gram‐positive spore‐forming rod‐shaped intestinal bacterium, pathogenic for many insect species, related to the phylum Firmicutes. Because of the presence of δ‐endotoxins, *B. thuringiensis* can serve as an insecticidal agent. Based on this feature, *B. thuringiensis* is used worldwide as a source of biopesticides for the control many insect species, especially Lepidoptera species but also Coleoptera and Diptera species (Bravo, Likitvivatanavong, Gill, & Soberon, 2011; Garczynski & Siegel, 2007). Consequently, *B. thuringiensis* is an important agent that can induce artificial declines in the population density of herbivores experiencing outbreaks and the understanding of the fundamental aspects of interaction between this bacteria and insects is needed for the effective applying of biocontrol against insect pests. Thus, the first question addressed in the current work is how phenological asynchrony affects the resistance of *L. dispar* to *B. thuringiensis*.

The level of insect resistance to *B. thuringiensis* as well as to many other pathogens is determined by the status of its innate immunity (Dubovskiy et al., 2016; Ma, Sarjan, Preston, Asgari, & Schmidt, 2005). One of the main parameters mediating the outcome of insect–bacteria interactions is the prevalence of lysozyme and lower molecular weight antimicrobial peptides (AMPs) (Hwang & Kim, 2011; Johnston & Rolff, 2015). Lysozymes are enzymes (EC 3.2.1.17) that catalyze the hydrolysis of the β‐1,4 glycosidic linkage between N‐acetyl muramic acid and N‐acetylglucosamine in the peptidoglycan of the cell wall of Gram‐positive bacteria, and it is widely distributed among vertebrates and invertebrates (Jolles & Jolles, 1984), whereas AMPs are short peptides that can destroy bacterial cells via several mechanisms (Bulet, Stocklin, & Menin, 2004). Detoxifying enzymes, such as nonspecific esterases, have been shown to represent an important midgut defense factor against *B. thuringiensis* toxins (Gunning, Dang, Kemp, Nicholson, & Moores, 2005). Thus, the second question addressed in the current work is how asynchrony affects the immune parameters of defoliators that are involved in antibacterial resistance.

One important finding related to the *B. thuringiensis*‐induced pathogenesis of hosts is that the pathogenic effect of *B. thuringiensis* on lepidopteran species, including *L. dispar*, is dependent on the presence of other species of nonpathogenic bacteria in the insect gut (Broderick et al. 2006, Broderick et al., 2009). However, there are several pieces of evidence indicating that the nonpathogenic gut microbiota is not required for the pathogenicity of *B. thuringiensis* (i.e., van Frankenhuyzen, Liu, & Tonon, 2010; Johnston & Crickmore, 2009; Raymond et al., 2009). Despite the absence of a general consensus, it is clear that the gut microflora community is not constant, even for the same herbivore species (i.e., Broderick et al. 2006, Broderick et al., 2009; Broderick, Raffa, & Handelsman, 2010 vs. van Frankenhuyzen et al., 2010). Recent studies have confirmed that the abundance of certain bacteria in the gut consortia of the same host species is dependent on the secondary metabolites present in the consumed food (Mason, Couture, & Raffa, 2014; Mason & Raffa, 2014; Mason, Rubert‐Nason, Lindroth, & Raffa, 2015). Thus, we predict that the composition of the gut bacterial community, which is dependent on the host plant allelochemicals, might contribute to insect resistance to *B. thuringiensis*. Over the past decade, a new and informative method for investigating bacterial communities has been developed. This method involves the metagenomic study of hypervariable fragments of 16S ribosomal RNA using high‐throughput sequencing (Armougom & Raoult, 2008; Chakravorty, Helb, Burday, Connell, & Alland, 2007). An important advantage of this method is the detection of uncultivated bacteria, which can be used to produce an overall estimate of the structure of the bacterial community. However, to the best of our knowledge, the study of *B. thuringiensis* pathogenesis using this informative approach has not been performed with the exception of a recent study of nonherbivorous species (Dubovskiy et al., 2016). Consequently, the third question addressed in the current work is how the structure of the bacterial community in the midgut of noninfected and *B. thuringiensis*‐infected larvae might change under the effect of phenological asynchrony between *B. pendula* and *L. dispar*. Thus, our study will ultimately provide insights into the main aspects of the interaction between *L. dispar* and *B. thuringiensis* under the synchronous and asynchronous development of the larvae and host plant and will clarify certain mechanisms that drive this interaction.

## 2 Methods

### 2.1 Study site and species

The experiments were conducted in the summer of 2013 at the Karasuk Research Station of the Institute of Systematics and Ecology of Animals, Siberian Branch RAS, Western Siberia, Russia (53°42′N 77°45′E). In these experiments, young artificially planted *B. pendula* trees (~10 m tall) were used as the host plant for *L. dispar* larvae. The trees were not noticeably attacked by wild defoliators during the 7 years prior to the experiments, and the natural density of gypsy moth egg masses ranged from 0 to 0.05 egg masses per tree. Gypsy moth eggs for the current study were collected in the autumn of 2012 from birch trees in the neighboring area (53°91′N 77°77′E) when the moth populations were in the increasing (pre‐peak) phase of their population cycle. The eggs were overwintered in a refrigerator at 4°C and used to provide a stock of experimental animals for the experiments conducted the following spring.

### 2.2 General experimental design

**Figure 1:**
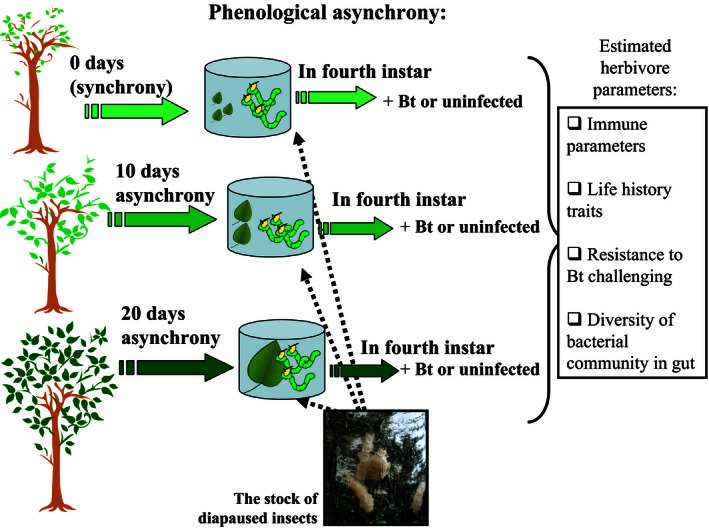
Experimental design of the simulated asynchrony between the hatching of *Lymantria dispar* larvae and the bud burst of *Betula pendula* and the subsequent larval infection with *Bacillus thuringiensis* (Bt)

The principal design of the experiment was the same as that used in our previous study (Martemyanov, Pavlushin, Dubovskiy, Yushkova, et al., 2015), and a detailed description can be found in our previous paper. The general design is also presented in Fig. 1. Briefly, to simulate the asynchrony between the development of *L. dispar* larvae and *B. pendula* foliage, we modified (delayed) the hatching time of the larvae. The rearing of hatched larvae was performed in the laboratory on natural leaves of *B. pendula* at 22 ± 1°C under a regime of natural daylight. All larvae were used from the same stock, kept in the refrigerator (Martemyanov, Pavlushin, Dubovskiy, Yushkova, et al., 2015). To feed the larvae, we deliberately selected trees of similar phenological states to minimize variations in budding between replicates. Two groups of trees were used to feed the two corresponding groups of experimental larvae: One group (five trees; group #1) was used to rear the larvae intended for the biochemical and molecular assays of the infected and uninfected insects (i.e., lytic activity, esterase activity, expression of the AMP genes, gut bacterial community); the second group (three trees; group #2) was used to study the survival rate of insects after *B. thuringiensis* infection. Two groups of trees were used to prevent high level of artificial defoliation within the same tree. Final level of artificial defoliation was not exceeded 50%. According to our previous data, this level of artificial defoliation does not induce the resistance of *B. pendula* against *L. dispar* (Bakhvalov, Bakhvalova, Morosova, & Martemyanov, 2006). To exclude the effect of individual features of the tree (i.e., variability among individual trees in leaf chemical composition; see Laitinen, Julkunen‐Tiitto, & Rousi, 2000), we used the same tree to investigate different points of asynchrony. Thus, each experimental tree was used to study all levels of asynchrony.

We used the following range of phenological mismatches between the birch trees and gypsy moths (the length and width of *B. pendula* leaves are provided in parentheses ± *SE*): “0 days of asynchrony” (synchronization point), larvae hatching was synchronized with the experimental tree budding (2.43 ± 0.18 cm, 2.31 ± 0.13 cm); “10 days of asynchrony,” larvae were hatched 10 days after the synchronization point (4.58 ± 0.09 cm, 3.94 ± 0.14 cm); “20 days of asynchrony,” larvae were hatched 20 days after the synchronization point (5.58 ± 0.11 cm, 4.96 ± 0.18 cm) (Fig. 1). To measure the expression of AMP genes in the midgut (see below), we used two intermediate points of asynchrony: “5 days of asynchrony” (3.99 ± 0.21 cm, 3.55 ± 0.21 cm) and “15 days of asynchrony” (5.42 ± 0.19 cm, 4.66 ± 0.10). This wide range of asynchrony was used because the temperature threshold of the spring development of *B. pendula* is close to −1 to 2°C (Rousi & Pusenius, 2005), while the temperature threshold of the spring development of *L. dispar* before hatching is higher (about 6°C) (Il'ynskiy & Tropin, 1965). Thus, under the strong continental climate in Western Siberia characterized by unstable spring weather with often fall of temperature, the significant delay of larvae development is often occurred. On the other hand, we never register the emergence of first instar *L. dispar* larvae before leaf opening.

Based on this experimental design, two possible options were available with respect to the mode of action of phenological asynchrony on insects: variation in the events related to leaf phenology (i.e., foliage quality, characteristics of the bacterial community on the leaf surface); and variation in the events related to the prolonged duration of egg diapause. However, we note the insignificant effect of the second option for the following reasons:

1. *L. dispar*, even after hatching, is well adapted to conditions such as starvation when the temperature is below 10°C (Hunter, 1993); in our case, when the eggs underwent diapause at 4°C, the effect of prolongation was minimal;
2. The emergence rate of the larvae was not significantly affected by the delay in hatching (Martemyanov, Pavlushin, Dubovskiy, Yushkova, et al., 2015); and
3. The range of studied delays of hatching is consistent with the naturally occurring variation in the hatching times of *L. dispar* larvae in the forests of Siberia (Martemyanov V.V., personal observation).

Thus, we believe that the main trigger driving the features of insects under the studied conditions is the dynamics of the host plant features during maturation or any other events that pertain to leaf development except (or at least to a much lesser degree) the delay of diapause in insect groups. We will focus more on this statement in our further discussion.

#### 2.2.1 *L. dispar* challenged by *B. thuringiensis*

The peroral larval challenge was performed with *B. thuringiensis* ssp. *kurstaki* collected from the museum of the Laboratory of Insect Pathology. One day after molting, fourth‐instar larvae were used for the inoculation. The concentration of *B. thuringiensis* spores and the crystal mixture (1:1) used for the inoculation was 5 × 10^7^ CFU/ml of water. The suspension was evenly applied on the leaf surface with a brush. Approximately 20 leaves were treated per 50 larvae used. *B. thuringiensis* possess a strong antifeedant effect; therefore, this amount of leaves was sufficient to induce *B. thuringiensis* infection, and a shortfall in the amount of infected food was not observed. The same amount of leaves treated with water was used as the control. We used 50 larvae per asynchrony point per concentration per tree, reared in cohort in 20‐L container, to estimate the true resistance against *B. thuringiensis*. The resistance of insects was estimated as the survival rate at two time points: up to 5 days after inoculation (to estimate the effect of *B. thuringiensis* because it is the rapidly progressing infection) and from inoculation to adult emergence (to estimate both effects: asynchrony, required for more continuous time periods, and *B. thuringiensis* together). Moreover, we also compared both the pupal weight and the residual duration of the larval stage after infection in the insects that survived *B. thuringiensis* infection to study the effect of bacterial disease in more detail.

### 2.3 Nonspecific esterases activity measured in the *L. dispar* midgut

The nonspecific esterases as well as all the following parameters were measured in the fourth‐instar larvae 1 day after inoculation with *B. thuringiensis* (2 days after molting in the fourth instar). Ten larvae per tree per treatment were used. For the enzyme assays, larval midguts from each group were dissected in ice‐cold potassium phosphate buffer (0.2 mol/L, pH 7.8, with 1 mmol/L EDTA) and ground in an ultrasound tissue homogenizer. The esterase activity in the midgut was measured using *p*‐nitrophenyl acetate as a substrate. The reaction mixture (1 ml) consisted of 20 μl of 0.01 mol/L p‐nitrophenyl acetate in 200 mmol/L phosphate buffer (pH 7.2) (Prabhakaran & Kamble, 1995). The esterase activity was measured as the difference in absorbance at 410 nm after 30 min of incubation with the substrate at 28°C.

### 2.4 Lysozyme‐like activity measured in the *L. dispar* midgut and hemolymph plasma

Lysozyme‐like activity was measured in the hemolymph and homogenized midgut. A total of 5 μl of hemocyte‐free hemolymph/supernatant of sonicated midgut tissue was mixed with phenylthiourea crystals, and 2 μl of the mixture was used to estimate the lysozyme‐like activity against the bacterium *Micrococcus lysodeikticus* (Lee, Cory, Wilson, Raubenheimer, & Simpson, 2006). Samples were placed into holes in agar medium containing the test microorganism, with the hemolymph samples incubated for 24 hr at 28°C and the midgut samples incubated for 48 hr at 28°C. Data were recorded as the area of the lytic zones, which were photographed using a digital camera (Canon, Japan) and then measured with Image Pro software (Media Cybernetics, Silver Spring, MD, USA) (Martemyanov, Dubovskiy, Belousova, et al., 2012; Martemyanov, Dubovskiy, Rantala, et al., 2012). Standard curves were obtained from a serial dilution of hen egg‐white lysozyme equivalents.

### 2.5 Amp expression measurements in the *L. dispar* midgut

We used five levels of phenological asynchrony for this experiment. Five insects per tree were used. The midguts were dissected in PBS and then cleaned and washed to separate the content of the gut lumen. After dissection, the guts were immediately prefrozen in liquid nitrogen and then stored at −80°C until further analysis. The midgut tissue was homogenized using a polyurethane pestle under strong freezing in liquid nitrogen. Five midguts from each tree were pooled together, and this pooled sample was used as a biological replicate. Three technical replicates of each of five biological replicates (five trees) were used per treatment.

Total RNA was isolated using the TRI Reagent (catalogue Nr T9424, Sigma, USA). The RNA solution was treated with DNase (catalogue Nr M6101, Promega, USA), and the concentration was estimated with a spectrophotometer (8453, Agilent, USA) and a nanovette (A44100‐AA, Beckman coulter, Germany). The RNA integrity was estimated by electrophoresis. A one‐step quantitative real‐time (qRT) PCR assay was conducted using a CFX96 Touch™ Real‐Time PCR Detection System (Bio‐Rad, USA). Each qPCR consisted of 50 mmol/L Tris–HCl at pH 8.3, 75 mmol/L KCl, 0.3 mmol/L each of dNTPs, 3 mmol/L MgCl_2_, 4 mmol/L DTT, M‐MuLV–RH reverse transcriptase, HS‐Taq DNA‐polymerase, SYBR Green, 0.3 μg diluted RNA, and 0.1 μmol/L of each primer in a total volume of 25 μl. The reactions were performed in triplicate to ensure consistent technical replication and run in 96‐well plates under the following conditions: 45°C for 30 min, 95°C for 10 min, and 40 cycles of 95°C for 30 s and 60°C for 1 min. The melting curves (60°C to 95°C) were derived for each reaction to ensure a single product. The amplification efficiency was assessed for each primer set using 10‐fold serial dilutions of RNA template. The mean quantification cycle (*C*_q_) values were calculated from the triplicate reactions; normalized against the geometric mean of two reference genes, 18S rRNA and the elongation factor‐Tu gene; and transformed to provide fold differences according to the formula $2^{-\Delta \Delta C_{q}}$ (Livak & Schmittgen, 2001).

The primer set for 18S rRNA was obtained from Dubovskiy, Whitten, Kryukov, et al. (2013), Dubovskiy, Whitten, Yaroslavtseva, et al. (2013), and the factor elongation‐Tu and gloverin precursor genes primers were obtained from Sparks, Blackburn, Kuhar, and Gundersen‐Rindal (2013). RNA‐seq data from Sparks et al. (2013) were assembled using Trinity software (Grabherr et al., 2011). BLAST searches for homologues of the known lepidopteran defensin and moricin mRNAs were subsequently performed to obtain the templates to design the corresponding *def* and *mor* primers using of the primer‐BLAST suite. The primer sequences are provided in Table S1. Studied AMP genes were set according to its ability to increase the expression level in *L. dispar* midgut under *B. thuringiensis* infection (Sparks et al., 2013).

### 2.6 16S rRNA metagenomic sequencing and data analysis

Three larvae per tree and five trees per treatment were used in this assay. The larvae midguts along with the gut contents were dissected and immediately frozen in liquid nitrogen. We then pooled three samples from the same tree in one sample. Accordingly, there were five biological replicates per treatment. Total DNA was extracted using the QIAamp DNA Stool Mini Kit (QIAGEN) according to the manufacturer's instructions.

The V3‐V4 region of the 16S rRNA genes was amplified with the primer pair 343F (5′‐CTCCTACGGRRSGCAGCAG‐3′) and 806R (5′‐GGACTACNVGGGTWTCTAAT‐3′) and combined with Illumina adapter sequences, a pad and a linker for two bases, and barcodes were attached to the primers (Caporaso et al., 2012). PCR amplification was performed in 50‐μl reactions containing 0.7 U Phusion Hot Start II High‐Fidelity, 1 ×  Phusion GC buffer (Thermo Fisher Scientific), 0.2 μmol/L of each forward and reverse primer, 10 ng of the template DNA, 2.3 mmol/L MgCl_2_ (Sigma‐Aldrich), and 0.2 mmol/L dNTP (Life Technologies). The thermal cycling conditions were as follows: initial denaturation at 98°C for 1 min, followed by 30 cycles of 98°C for 15 s, 62°C for 15 s, and 72°C for 15 s, with a final extension at 72°C for 10 min. A total of 200 ng PCR product from each sample was pooled together and purified using a MinElute Gel Extraction Kit (Qiagen). The sample libraries for sequencing were prepared according to the MiSeq Protocol (Illumina) and previously described protocols (Caporaso et al., 2011, 2012). Sample denaturation was performed by mixing 4.5 μl of the combined PCR products (4 nmol/L) and 4.5 μl 0.2 mol/L NaOH. Denatured DNA was diluted to 14 pmol/L, and 510 μl of this DNA was mixed with 90 μl of the 14 pmol/L Phix library. A total of 600 μl of the sample mixture was loaded along with the customized sequencing primers for the forward, reverse, and index reads into the corresponding wells on the reagent cartridge of the 500‐cycle PE kit and run for 2 × 250 bp paired‐ends sequencing on a MiSeq Illumina sequencer at the SB RAS Genomics Core Facility (ICBFM SB RAS, Novosibirsk, Russia).

The raw sequences were analyzed with the UPARSE pipeline (Edgar, 2013) using USEARCH v8.1.1756. The UPARSE pipeline performed paired read merging; read quality filtering; length trimming; identical read merging (dereplication); singleton read removal; chimera removal; and OTU clustering using the UPARSE‐OTU algorithm. The OTU sequences were assigned a taxonomy using the RDP classifier 2.11 (Wang, Garrity, Tiedje, & Cole, 2007). Community structure analyses were performed based on the phylum and genus taxonomy levels. The microbial community diversity (Shannon index) was calculated in Explicet 2.10.5 (Robertson et al., 2013) at the rarefaction point with 500 bootstrap resamplings.

One replicate of the gut sample was significantly contaminated by *Spiroplasma*, which was not detected in any of the other studied samples. Accordingly, this replicate was excluded from the experiment. Most of the samples contained an average of approximately 70% sequences associated with chloroplast 16S rRNA, and these sequences were not considered in the analysis.

### 2.7 Statistical analysis

The effects of phenological mismatches and *B. thuringiensis* infection on the pupal weight, development time, immune parameters, and detoxification system were tested using a mixed model analysis (SPSS 19.0 for Windows), in which the random factors were tree, tree*mismatch, tree*infection, and tree*mismatch*infection, and the fixed factors were mismatch, *B. thuringiensis* infection, and interactions. If certain random factors were redundant items (when covariance parameters were estimated), they were omitted from the final model. We used the restricted maximum‐likelihood method to estimate the parameters, and the type III sum of squares was selected (Martemyanov, Dubovskiy, Rantala, et al., 2012). To study the effects of the phenological mismatches and *B. thuringiensis* infection in more detail, pairwise contrasts were tested with the LSD method. We used a two‐way ANOVA followed by the post hoc Fisher LSD procedure to calculate the data on the survival rate of infected insects and calculate the expression of the AMP genes (a one‐way ANOVA was used for the gloverin precursor gene). The dominant OTUs were separately compared by a two‐way ANOVA followed by the post hoc Fisher LSD procedure. Shannon H indexes were also compared via a two‐way ANOVA followed by a post hoc Fisher LSD procedure. The larval development rate and AMP gene expression data were log10‐transformed prior to the analyses to meet the assumptions of the parametric tests. All the data represented in percentages prior to the analysis were transformed using the arcsine of the square root.

## 3 Results

### 3.1 Insects challenged by *B. Thuringiensis*

**Figure 2:**
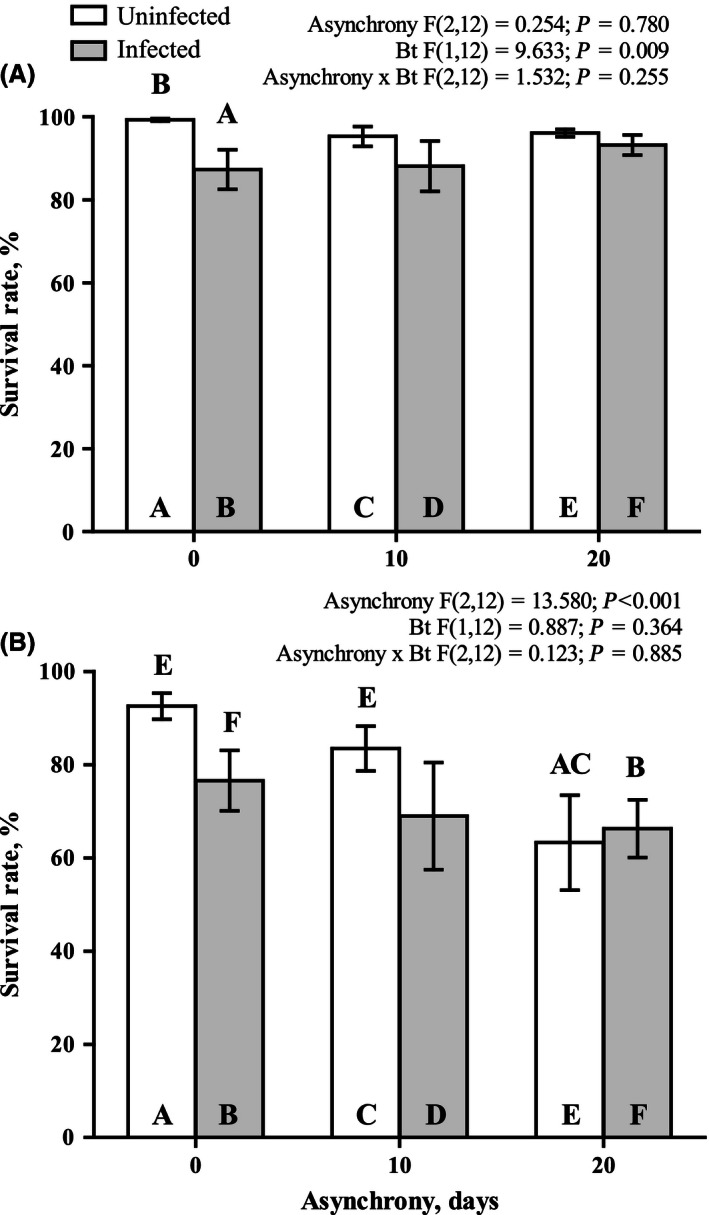
Effects of asynchrony and *Bacillus thuringiensis* infection on the survival rate of *Lymantria dispar* measured within 5 days (A) postinfection and throughout the entire premature (B) postinfection period. The results of the ANOVA are provided above the figure. The results of pairwise comparisons (Fisher LSD tests) are indicated with letters. The letters above the bar indicate significant differences (at *p* < .05) compared with the bars identified by the same letters within the bar

**Figure 3:**
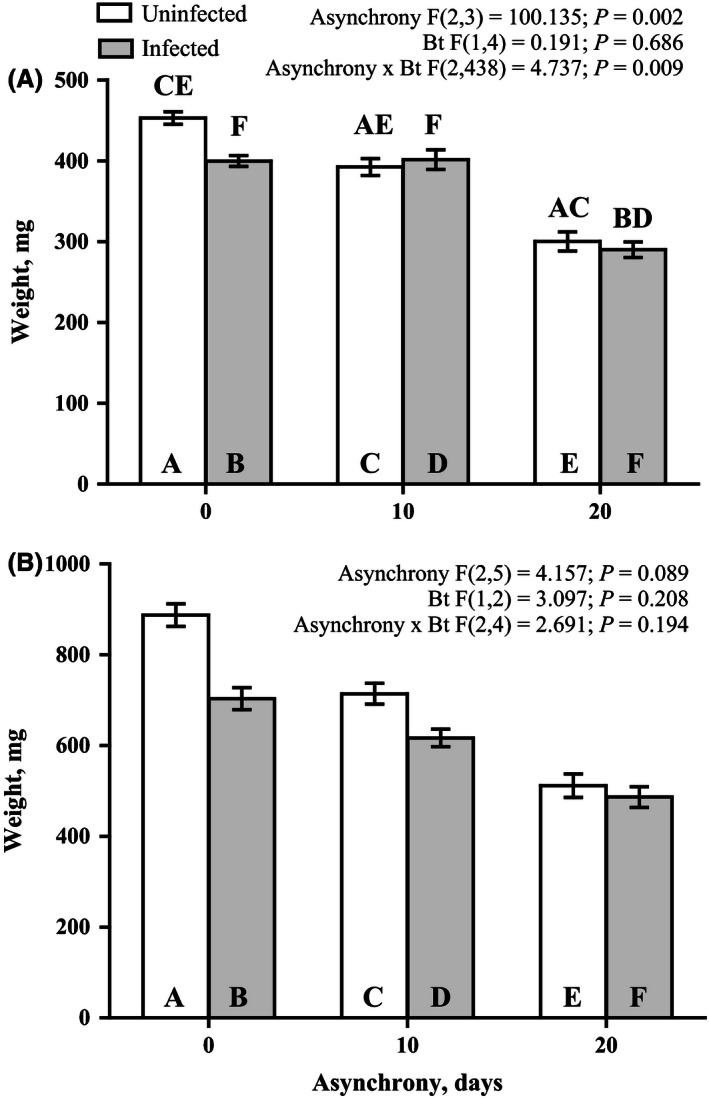
Effects of asynchrony and *Bacillus thuringiensis* infection on the pupal weight of surviving male (A) and female (B) *Lymantria dispar* after infection. The results of a mixed model analysis are provided above the figure. The results of pairwise comparisons (Fisher LSD tests) are indicated by letters. The letters above the bar indicate significant differences (at *p* < .05) compared with the bars identified by the same letters within the bar

**Figure 4:**
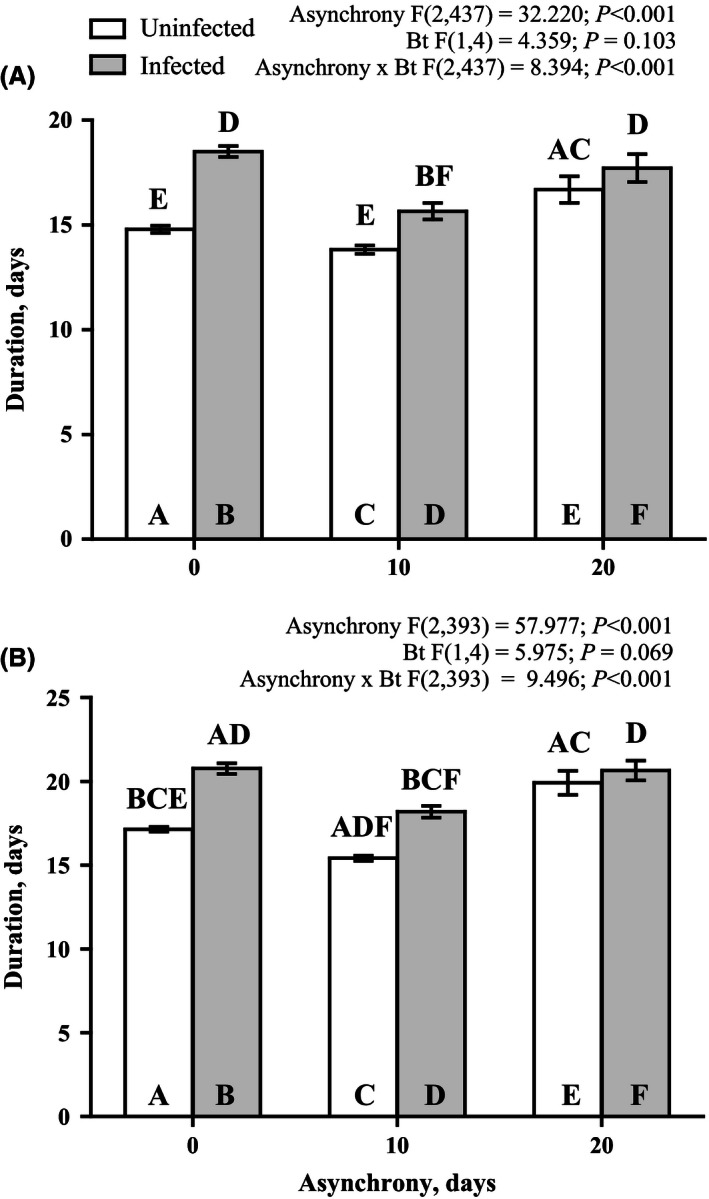
Effects of asynchrony and *Bacillus thuringiensis* infection on the prepupal development time after larval inoculation of surviving males (A) and females (B) of *Lymantria dispar* after infection. The results of a mixed model analysis are provided above the figure. The results of pairwise comparisons (Fisher LSD tests) are indicated by letters. The letters above the bar indicate significant differences (at *p* < .05) compared with the bars identified by the same letters within the bar

The survival rate data demonstrate that *B. thuringiensis* effect was significant only within 5 days after larvae infection (Fig. 2A), while the effect of asynchrony was found only throughout the entire premature postinfection period (Fig. 2A). Pairwise comparison of the survival rate between infected and noninfected larvae indicates the significant decrease in survival only for synchronously reared insects (Fig. 2A). There was found the interaction between studied factors when we record the life history traits of the surviving after *B. thuringiensis* infection larvae (pupal weight and residual duration of the larval stage after infection) excepting female pupae weight (Figs 3 and 4).

### 3.2 Lysozyme‐like and nonspecific esterase activity measured in *L. dispar* larvae

**Figure 5:**
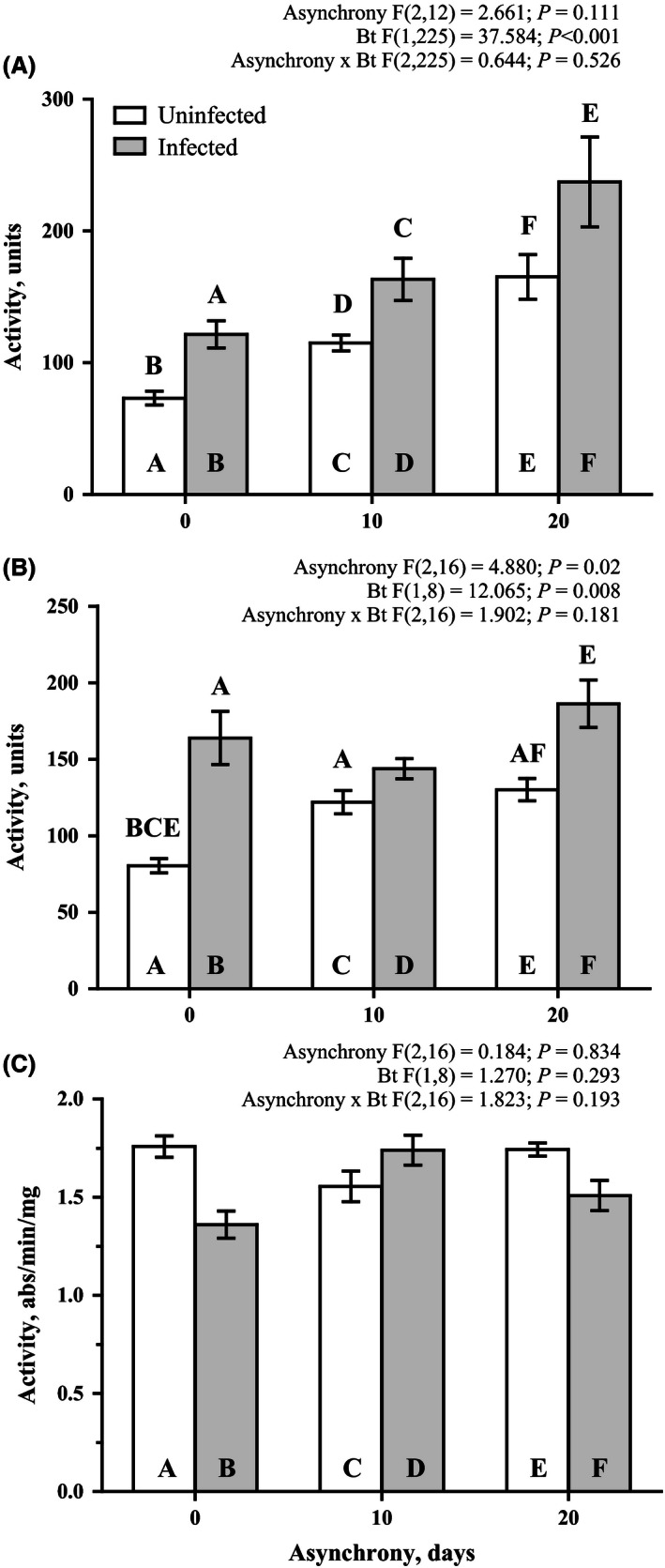
Effects of asynchrony and *Bacillus thuringiensis* infection on the lytic activity in hemolymph plasma (A), lytic activity in the midgut tissue (B), and nonspecific esterase activity in the midgut tissue (C) of *Lymantria dispar* fourth‐instar larvae on the first day after infection. The results of a mixed model analysis are provided above the figure. The results of pairwise comparisons (Fisher LSD tests) are indicated by letters. The letters above the bar indicate significant differences (at *p* < .05) compared with the bars identified by the same letters within the bar

Lysozyme‐like activity was induced by bacterial infection in both the plasma of the hemolymph and midgut tissue at all the studied rates of asynchrony with the exception of the 10‐day asynchrony in relation to the midgut lysozyme‐like activity (Fig. 5A,B). Both the basic and induced levels of lysozyme‐like activity in the plasma of the hemolymph were tend to be affected by the asynchrony, with larvae that developed asynchronously with the host plant leaves possessing higher lysozyme‐like activity (Fig. 5A) than the larvae that developed synchronously. Moreover, the lysozyme‐like activity in the plasma of the hemolymph was linearly dependent on the asynchrony level (*r* = .998, *p* = .033 for the basic level and *r* = .983, *p* = .1165 for the *B. thuringiensis*‐induced level). The assay of esterase activity in the midgut did not reveal strong patterns among the characteristics of the asynchronously developed larvae infected by *B. thuringiensis* (Fig. 5C).

### 3.3 Amp genes expression measurement in *L. dispar* larvae

**Figure 6:**
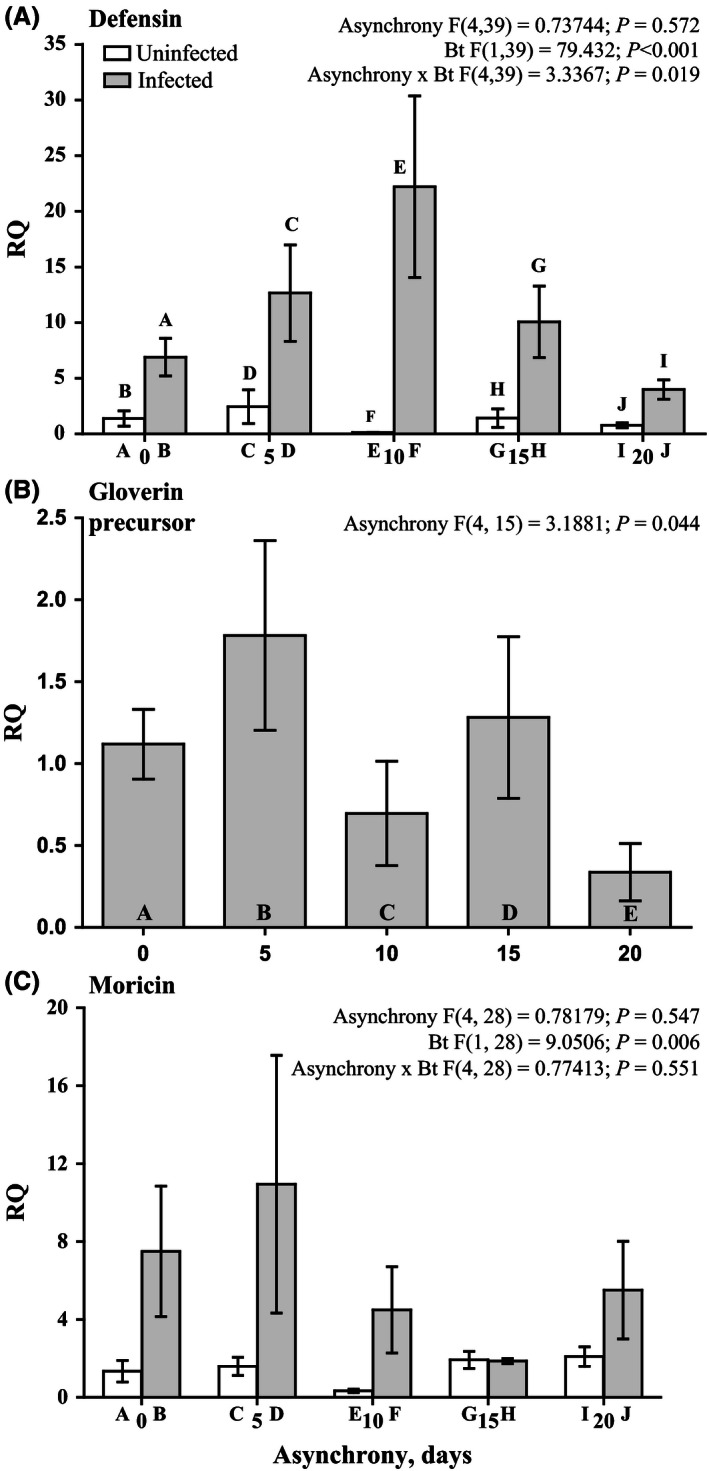
Effects of asynchrony and *Bacillus thuringiensis* infection on the expression level of defensin (A), gloverin precursor (B), and moricin (C) genes in the midgut tissue of *Lymantria dispar* fourth‐instar larvae on the first day after infection. The data are transformed to provide fold differences according to the formula $2^{-\Delta \Delta C_{q}}$. The results of a mixed model analysis are provided above the figure. The results of pairwise comparisons (Fisher LSD tests) are indicated by letters. The letters above the bar indicate significant differences (at *p* < .05) compared with the bars identified by the same letters within (or below) the bar. The *y*‐axis (A, C) represents the expression in uninfected/infected larvae as a fold change relative to the uninfected larvae from the synchrony point (i.e., 0 days of asynchrony). There was no expression of gloverin precursor in the midgut of noninfected larvae. Therefore, the *y*‐axis (B) represents the expression in infected larvae as a fold change relative to the infected larvae starting from the synchrony point (i.e., 0 days of asynchrony)

The analysis of AMP genes, including moricin, defensin, and gloverin precursor, in the insect midgut revealed that the expression of these genes was upregulated under the *B. thuringiensis* treatment (Fig. 6A–C). Moreover, gloverin precursor was only expressed at detectable levels after larval infection with *B. thuringiensis* (Fig. 6B). Asynchrony did not produce a consistent effect on the expression rate of studied AMPs genes (Fig. 6A–C), while significant interaction between asynchrony and *B. thuringiensis* infection was found for the expression of defensin gene (Fig. 6A).

### 3.4 Gut bacterial community measured in the *L. dispar* larvae

The analysis of the v3‐v4 16S rRNA region of the *L. dispar* gut bacterial community identified 203 OTUs, and this number varied greatly. The range of copy numbers varied between 3,300 and 61,000 copies per treatment after the removal of chimaeras and OTUs classified as plastids.

**Figure 7:**
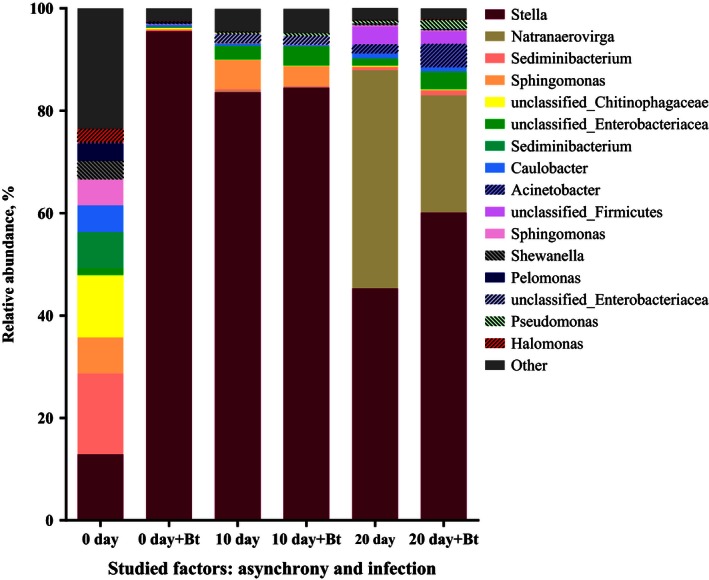
Effects of asynchrony and *Bacillus thuringiensis* infection on the diversity of the gut bacterial community. OTUs are classified at the genus level. “Bt” in *x* axis means challenge with *Bacillus thuringiensis*

**Figure 8:**
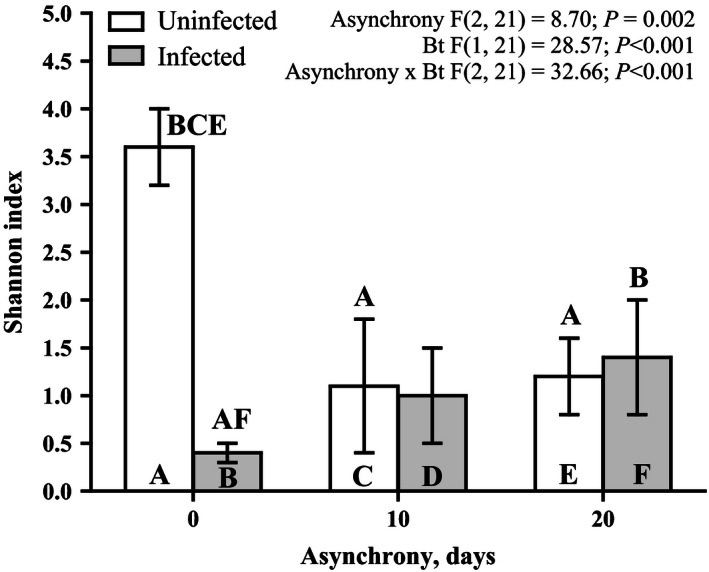
Shannon indexes. Comparisons were performed via a two‐way ANOVA (the results of ANOVA are provided above the figure) followed by a post hoc Fisher LSD test. The letters above the bar indicate significant differences (at *p* < .05) compared with the bars identified by the same letters within the bar

**Figure 9:**
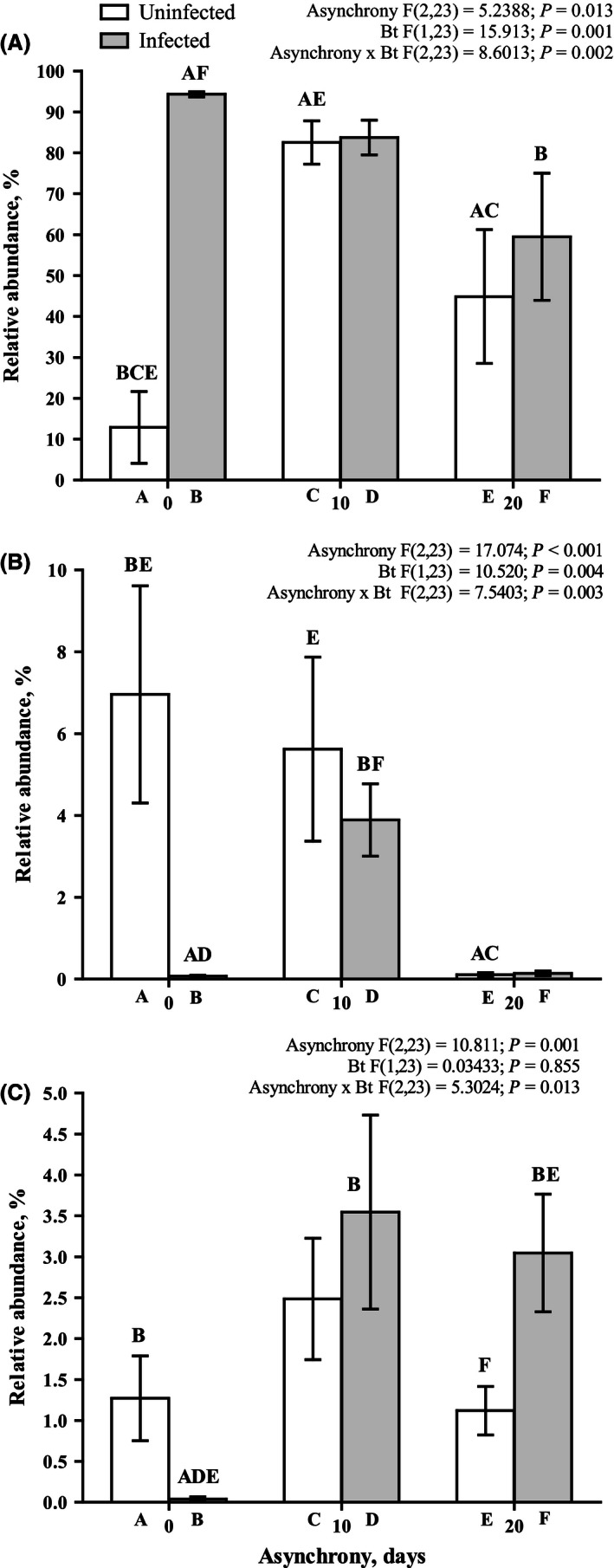
Effects of asynchrony and *Bacillus thuringiensis* infection on the relative abundance of OTUs classified as Stella (A), Sphingomonas (B), and unclassified Enterobacteriaceae (C) in the gut of *Lymantria dispar* fourth‐instar larvae on the first day after infection. The results of the ANOVA are provided above the figure. The results of pairwise comparisons (Fisher LSD tests) are indicated by letters. The letters above the bar indicate significant differences (at *p* < .05) compared with the bars identified by the same letters below the bar

Only the larvae developing synchronously with *B. pendula* leaves possessed a sufficiently high diversity of bacterial taxa, and the Shannon's diversity index for this group had the highest value observed in the study (Figs 7, 8 and S1). When the larvae developed asynchronously with *B. pendula* leaves, the diversity of bacteria was significantly reduced and only included one (in the case of 10‐day asynchrony) and two (in the case of 20‐day asynchrony) dominant OTUs, which were unclassified by RDP (unclassified bacteria and unclassified Clostridiales). A BLAST analysis identified *Stella humosa* and *Natranaerovirga hydrolytica* as the species with maximal identity values of 77.8% (E‐value = 3.91E‐86) and 91.4% (E‐value = 2.63E‐164), respectively (Fig. 7). *B. thuringiensis* infection decreased the diversity of the bacterial community only in the guts of the synchronously reared larvae (Figs 7 and S1) and did not affect the diversity of the bacterial flora of the asynchronous larvae. A detailed comparison of the dominant and subdominant OTU representatives reveals the following information. The relative abundance of the OTUs close to *Stella humosa* was dramatically increased by the infection and asynchronous rearing of insects (Figs 7 and 9A), whereas the relative abundance of the OTUs for two representatives of *Sediminibacterium* and an unclassified representative of the family Chitinophagaceae was dramatically decreased by the infection and asynchronous rearing of insects (Fig. 7, ANOVA results for Sediminibacterium bars filled by rose color: asynchrony *F*_2, 23_ = 32.513, *p* < .001; bt *F*_1, 23_ = 40.65, *p* < .001; asynchrony*bt *F*_2, 23_ = 36.64, *p* < .001; ANOVA results for Sediminibacterium bars filled by green color: asynchrony *F*_2, 23_ = 18.31, *p* < .001; bt *F*_1, 23_ = 24.90, *p* < .001; asynchrony*bt *F*_2, 23_ = 20.84, *p* < .001; ANOVA results for Chitinophagaceae: asynchrony *F*_2, 23_ = 57.84, *p* < .001; bt *F*_1, 23_ = 51.50, *p* < .001; asynchrony*bt *F*_2, 23_ = 46.90, *p* < .001). The relative abundance of the OTUs identified as *Sphingomonas* declined in the larvae reared under the 20‐day asynchrony and the larvae reared under synchrony and infected by *B. thuringiensis* (Fig. 9B). A significant interaction was observed between asynchrony and *B. thuringiensis* infection for the relative abundance of unclassified Enterobacteriaceae in the insects (Fig. 9C). In particular, the challenge of synchronously reared insects by *B. thuringiensis* resulted in a significant decrease in the relative abundance of unclassified Enterobacteriaceae, whereas the challenge of asynchronously reared insects resulted in a significant increase in the relative abundance of these OTUs (Fig. 9C).

## 4 Discussion

In this study, we demonstrated for the first time how the phenological asynchrony between host plant and herbivorous insects affects the interaction between insects and bacterial disease. We found that the susceptibility of *L. dispar* larvae to *B. thuringiensis* peroral infection decreased in 5 days after infection when the larvae developed asynchronously with *B. pendula* leaves. Even if the larvae survived the *B. thuringiensis* infection, the larval developmental rate and also the pupae weight of infected larvae were similarly affected by the phenological asynchrony. *B. thuringiensis* infection results in negative effect of studied parameters only when the larvae were reared synchronously with their host plant. This finding is entirely inconsistent with the findings of our recent study in which the susceptibility of *L. dispar* larvae to another pathogen (specifically, *Lymantria dispar* multiple nucleopolyhedrovirus) was found to significantly increase by phenological asynchrony (Martemyanov, Pavlushin, Dubovskiy, Yushkova, et al., 2015). Thus, this result explicitly demonstrates that the effect of phenological asynchrony on insect–pathogen interactions is not uniform and depends on the type of pathogen.

For the first time, we demonstrated that asynchrony between insects and their host plants induces dramatic changes in the structure of the community of nonpathogenic midgut bacteria as well as the patterns of antibacterial defense (both local and systemic) of defoliators. This finding could significantly clarify subsequent interactions between insects and gut nonpathogenic bacteria after the herbivore is challenged with a pathogen. The gut nonpathogenic microflora of insects are known to modulate the innate antimicrobial immunity status of noninfected individuals in a manner similar to that in humans (reviewed in Douglas, 2014). In particular, Freitak and coauthors have clearly demonstrated that the presence of nonpathogenic bacteria in the gut lumen of *Trichoplusia ni* larvae triggers antibacterial defenses, including lytic activity and antimicrobial peptide gene expression (Freitak, Wheat, Heckel, & Vogel, 2007). Moreover, a trade‐off has been observed between bacterially triggered antibacterial defense (AMPs and lytic activity), phenoloxidase‐mediated defenses, and life history traits (Freitak et al., 2007), and this finding is consistent with our data obtained for *L. dispar* larvae (the data presented here and in Martemyanov, Pavlushin, Dubovskiy, Yushkova, et al., 2015) in which the lytic activity increased but the phenoloxidase activity and life history traits decreased when the larvae were reared asynchronously with the host plant's seasonal development. It is interesting that only the lytic activity in the larval midgut was increased by asynchrony, whereas the AMP gene expression was not regularly affected by asynchrony. Moreover, we did not observed transcripts for gloverin precursor in any of the noninfected larvae, indicating the inducible character of this gene for gypsy moths (Sparks et al., 2013; the data of the current study). Thus, although the same low molecular weight antibacterial peptides can be induced by different types of pathogens, including fungi (Dubovskiy, Whitten, Kryukov, et al. 2013; Dubovskiy, Whitten, Yaroslavtseva, et al. 2013), we show that the expression of AMP genes is less sensitive to noninfectious asynchrony‐related factors compared with the expression of genes encoding lysozyme‐like proteins in insects.

Seasonal changes during leaf development present two modes of action on the midgut bacterial community structure. First, dramatic changes occur in the leaf chemistry of *B. pendula* (including flavonoids, cinnamic acids, condensed tannins, triterpenoids, sterols, fatty acids, and amino acids) in the first 20 days after leaf budding (Chernyak et al., 2016; Martemyanov, Pavlushin, Dubovskiy, Yushkova, et al., 2015); thus, because the structure of the gut bacterial community is modified by the environmental conditions within the midgut lumen (Mason et al., 2014, 2015), significant changes in the chemicals of *B. pendula* leaves (and, consequently, the midgut lumen) would change the diversity of the bacterial community in the midgut of the larvae. Second, the dynamics of the bacterial community outside the insect gut (within the plant phyllosphere) are mediated by bacterial immigration, emigration, reproduction, and death (reviewed in Kinkel, 1997). *L. dispar* larvae obtain the members of their gut bacterial consortia from consumed leaves rather than their parents (Broderick, Raffa, Goodman, & Handelsman, 2004; Mason & Raffa, 2014). Most likely, the absolute (not relative) amount of phyllosphere‐associated bacteria will increase during the spring because of (i) cumulative reproduction and (ii) significant decreases in ultraviolet‐induced mortality related to shading caused by the increasing area of the growing leaf (Moise & Aynsley, 1999). Both of these reasons will inevitably increase competition between members of the bacterial community within the phyllosphere (Carter et al., 2012; Cooley, Chao, & Mandrell, 2006) and result in significant changes in the structure of the bacterial community after leaf consumption.

Our results show that when larvae feed on mature leaves, the negative effects of the entomopathogenic bacterium *B. thuringiensis* are eliminated. This event contributes to the effect of *B. thuringiensis* on the diversity of the gut bacterial community, and a significant effect of *B. thuringiensis* infection was only observed when the larvae fed on young leaves, whereas no effect of *B. thuringiensis* was observed when the larvae fed on mature leaves. *B. thuringiensis* produces special metabolites (bacteriocins) to compete with other symbiotic or opportunistic bacteria (reviewed in de la Fuente‐Salcido, Casados‐Vazquez, & Barboza‐Corona, 2013), which explains the decreased diversity of gut bacterial consortia after successful infection by *B. thuringiensis* (i.e., increased herbivore mortality rate) in synchronously developing larvae. The absence of this effect in asynchronously reared larvae may be related to the Gram‐negative bacteria contained in the gut of these insects because Gram‐negative bacteria are tolerant to *B. thuringiensis* bacteriocins (Cherif, Rezgui, Raddadi, Daffonchio, & Boudabous, 2008), or it may be related to the elimination of *B. thuringiensis* by the midgut immunity (lysozyme‐like activity). Moreover, the enhanced resistance to *B. thuringiensis* of larvae fed mature leaves could be related to other midgut conditions (plant metabolites or other leaf quality‐associated features) that result in the disruption of *B. thuringiensis* toxins (Griffitts & Aroian, 2005).

Our study provides new information on whether nonpathogenic gut bacteria are required for effective *B. thuringiensis* pathogenesis (Broderick et al. 2006, Broderick et al., 2009; Johnston & Crickmore, 2009; Raymond et al., 2009; van Frankenhuyzen et al., 2010). The collected data demonstrate that the gut bacterial community of the host is generally involved in the pathogenic effect of *B. thuringiensis*, although these interactions among the bacteria within the host gut are complicated.

In conclusion, our study demonstrates the involvement of nonpathogenic midgut bacteria in the plant–insect asynchrony‐mediated regulation of the interaction between insects and *B. thuringiensis*. Moreover, the data from the present study along with the findings of our previous study (Martemyanov, Pavlushin, Dubovskiy, Yushkova, et al., 2015) indicate that the effect of asynchrony on herbivore antibacterial defenses can be opposite to compare with herbivore antiviral defense due to differential effects of asynchrony on herbivore innate immune parameters. The ecological findings proposed within these two fundamental studies could also be highly applicable to the management of *L. dispar* populations because they show that *B. thuringiensis* or nucleopolyhedrovirus‐based products should be applied according to the synchronous or asynchronous development between larvae and its host plant. This approach will optimize the effectiveness of such products without requiring an increase in the applied dosage.

## Funding Information

Grant from the President of the Russian Federation, Russian Foundation for Basic Research, (Grant/Award Number: 12‐04‐01228, 15‐04‐08197, 15‐54‐45083), Russian Science Foundation, (Grant/Award Number: 15‐14‐10014)

## Conflict of Interest

None declared.

## Data Accessibility

The data will be archived in Dryad Digital Repository.
