# Supplementary material for: Phenological asynchrony between host plant and gypsy moth reduces insect gut microbiota and susceptibility to Bacillus thuringiensis
Source: Ecol Evol. 2016 Sep 22;6(20):7298–310. doi: 10.1002/ece3.2460 (PMC5513265; doi:10.1002/ece3.2460)
Supplement: Supplementary file 1 [file ECE3-6-7298-s001.doc]

Fig. S1.

The effect of both asynchrony and Bacillus thuringiensis infection on the diversity of lymantria dispar gut bacterial community. OTUs are classified at genus level.
