# Supplementary material for: Phenological asynchrony between host plant and gypsy moth reduces insect gut microbiota and susceptibility to Bacillus thuringiensis
Source: Ecol Evol. 2016 Sep 22;6(20):7298–310. doi: 10.1002/ece3.2460 (PMC5513265; doi:10.1002/ece3.2460)
Supplement: Supplementary file 2 [file ECE3-6-7298-s002.doc]

Table S1

Primer sequences used for qRT-PCR analysis

| Gene name | Forward primers 5’->3’ | Reverse primers 5’->3’ |
| --- | --- | --- |
| 18s rRNA | CACATCCAAGGAAGGCAG | AGTGTACTCATTCCGATTACGA |
| FETu | CGTCTCCTGAAGCCAATG | TGATAACTCCTGTGCCAAG |
| Gloverin precursor | GCATACACATACGCTCA | TGTAACCGCCTCTACCA |
| Defensin | ATGACTAACACAAATGCCGT | CAGTTACTATTCCTGGGCGT |
| Moricin | TGCTCTTCTAATCCCAACGA | AGCACGAAGTCCTTTACCAA |
